# Osteogenesis imperfecta—pathophysiology and therapeutic options

**DOI:** 10.1186/s40348-020-00101-9

**Published:** 2020-08-14

**Authors:** Julia Etich, Lennart Leßmeier, Mirko Rehberg, Helge Sill, Frank Zaucke, Christian Netzer, Oliver Semler

**Affiliations:** 1 Dr. Rolf M. Schwiete Research Unit for Osteoarthritis, Orthopedic University Hospital Friedrichsheim gGmbH, Frankfurt/Main, Germany; 2 University of Cologne, Faculty of Medicine and University Hospital Cologne, Institute of Human Genetics, Cologne, Germany; 3 Department of Pediatrics, Faculty of Medicine and University Hospital Cologne, University of Cologne, Kerpener Straße 62, Cologne, Germany; 4 Faculty of Medicine and University Hospital Cologne, Center for rare diseases, University of Cologne, Cologne, Germany

**Keywords:** Osteogenesis imperfecta, Pathophysiology, Genetic heterogeneity, Therapy, Bisphosphonates

## Abstract

Osteogenesis imperfecta (OI) is a rare congenital disease with a wide spectrum of severity characterized by skeletal deformity and increased bone fragility as well as additional, variable extraskeletal symptoms. Here, we present an overview of the genetic heterogeneity and pathophysiological background of OI as well as OI-related bone fragility disorders and highlight current therapeutic options.

The most common form of OI is caused by mutations in the two collagen type I genes. Stop mutations usually lead to reduced collagen amount resulting in a mild phenotype, while missense mutations mainly provoke structural alterations in the collagen protein and entail a more severe phenotype. Numerous other causal genes have been identified during the last decade that are involved in collagen biosynthesis, modification and secretion, the differentiation and function of osteoblasts, and the maintenance of bone homeostasis.

Management of patients with OI involves medical treatment by bisphosphonates as the most promising therapy to inhibit bone resorption and thereby facilitate bone formation. Surgical treatment ensures pain reduction and healing without an increase of deformities. Timely remobilization and regular strengthening of the muscles by physiotherapy are crucial to improve mobility, prevent muscle wasting and avoid bone resorption caused by immobilization. Identification of the pathomechanism for *SERPINF1* mutations led to the development of a tailored mechanism-based therapy using denosumab, and unraveling further pathomechanisms will likely open new avenues for innovative treatment approaches.

## Introduction

Osteogenesis imperfecta (OI) is a congenital disease which presents with a wide range of phenotypes. With a suspected incidence of 1:20,000, OI is a rare disease. Depending on its severity, affected individuals can live a mostly unrestricted, independent life, or they are severely impaired in their mobility, require a wheelchair, and may depend on the support of caregivers. The intellectual abilities are not impaired. In more than 80% of patients, the genetic defect is located in *COL1A1/2* and shows a dominant mode of inheritance. During the last decade, different forms of inheritance and numerous other causal genes have been described. The disease OI changed from a clearly defined clinical picture to a group of genetic diseases with the common characteristic of reduced bone stability as we reviewed recently in a german article [1]. Different classifications are currently used and focus either on the phenotype or on the underlying genetic defect. Recently, a classification based on the skeletal phenotype and deformities of the long bones has been introduced and might be helpful in the clinical setting in the future [2].

### Symptoms

The symptoms of the disease can be divided into skeletal and extraskeletal findings. Skeletal symptoms are the decreased bone mass leading to reduced bone stability. This results in an increased fracture rate of the long bones after inadequate trauma, as well as deformities of vertebrae. Scoliosis is an additional problem that develops frequently during puberty in more severely affected patients and can lead to an impairment of the pulmonary function. Short stature is present in almost all patients and extremities can be disproportioned. There may also be axis deviations and differences in length of the long bones.

As a collagen disorder, additional extraskeletal symptoms can include hypermobility of ligaments and increased fragility of vessels. An impact on heart valves has also been described as well as an early hearing loss. An obvious but not always persistent finding is a blue-gray discoloration of the sclera in approx. 50% of OI patients. Due to the close biochemical relationship between collagen and dentin, the teeth are affected in some patients leading to dentinogenesis imperfecta with amber-colored appearance and increased brittleness.

In summary, patients with OI present with a wide range of symptoms and with a high variability of their phenotype. This variability in clinical severity can only be explained partially by the causative gene and the type of mutation. Understanding the different pathways which cause the brittleness of the skeleton is the premise to develop new therapeutic options to improve the quality of life of these patients [3].

### Pathophysiology

#### Collagen genes

Heterozygous mutations in the genes *COL1A1* and *COL1A2* are the most common cause of OI [4]. The large size of the genes explains the numerous known mutations and part of the heterogeneity of the clinical symptoms. So far, no reliable genotype-phenotype correlation has been identified. Nevertheless, two different pathophysiologies can be described: Loss-of-function mutations like stop mutations lead to haploinsufficiency. Patients have a reduced amount of collagen, but this is of normal quality. In contrast, other mutations (mostly glycine substitutions) lead to qualitative alterations of the extracellular matrix, since the collagen molecules and later fibrils cannot assemble properly. This results in more severe clinical courses. The qualitative disturbance and the inadequate stability of the collagenous bone substance also stimulate bone resorption, since the body tries to break down the qualitatively disturbed bone. Due to the reduced bone stability and the stimulated degradation, osteoblasts produce as much osteoid as possible, but this is of lower quality. This results in a “high turnover osteoporosis”.

**Fig. 1:**
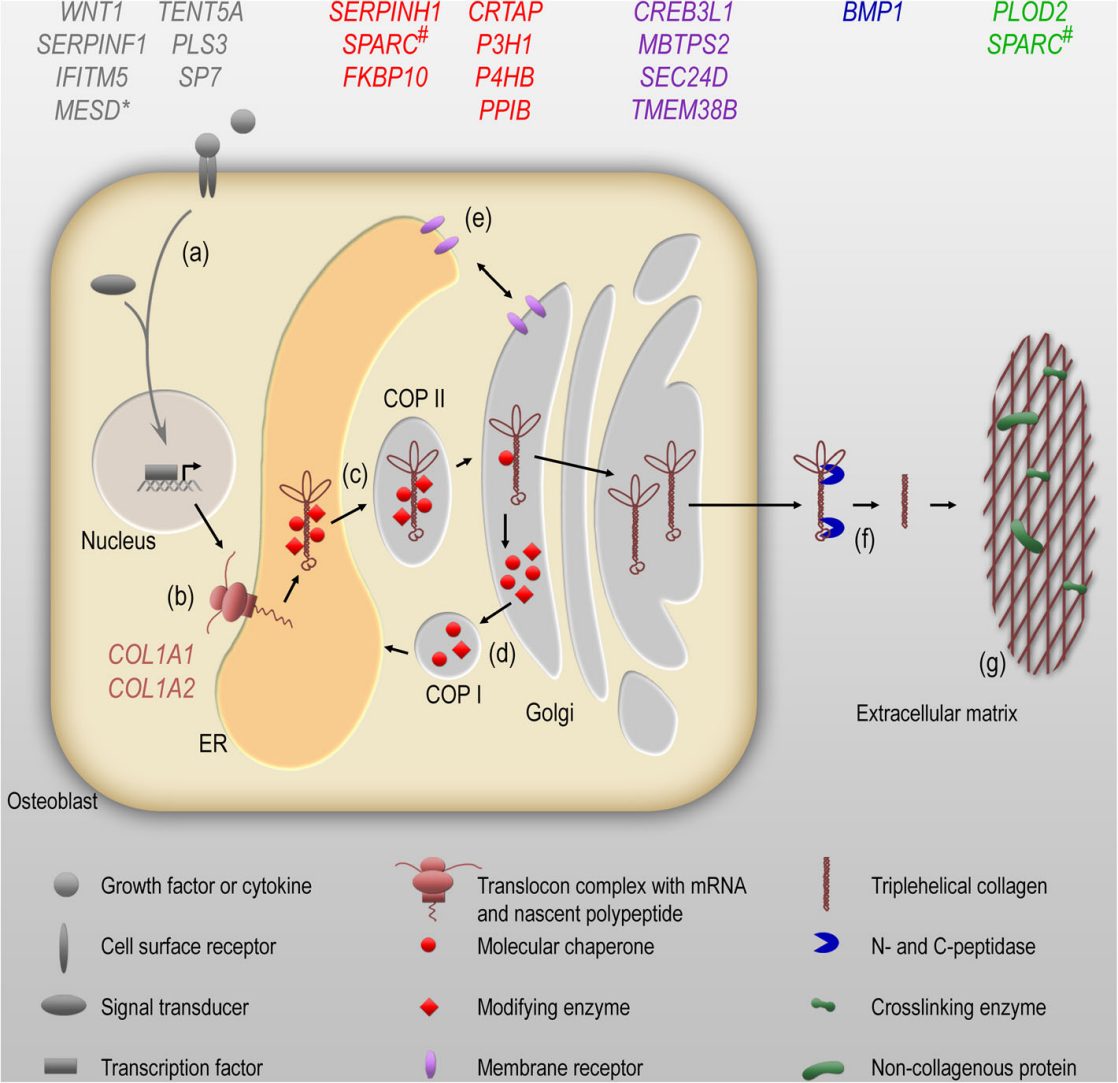
OI genes involved in collagen biosynthesis and maintenance of bone homeostasis. Growth factors and cytokine signal through cell surface receptors to initiate an intracellular signal cascade (**a**). The transduction of the signal results in the nuclear translocation of transcription factors to regulate the expression of genes involved in osteoblast differentiation or function and collagen biosynthesis. Several regulated genes encode proteins that upon secretion influence osteoclast formation and activity (RANKL, osteoprotegerin, sclerostin; not shown). The α1- and α2-chains of collagen type I, the main collagen produced in osteoblasts, are translated into the rough endoplasmic reticulum (ER) (**b**). Molecular chaperones support the folding of collagen chains enabling hydroxylation of proline and lysine residues by hydroxylases as well as subsequent glycosylation that is indispensable for proper formation of triplehelical collagen (**c**). The procollagen is secreted by a coat protein complex II (COP II)-mediated vesicular transport through the Golgi network into the extracellular space. Molecular chaperones and modifying enzymes dissociate pH-dependently from procollagen in the Golgi intermediate compartment and circulate COP I-mediated back to the ER (**d**). Integral proteins in the ER and Golgi membranes, such as ion channels or ER stress sensors, maintain the intracellular homeostasis and in that way preserve the secretory pathway (**e**). The N- and C-propeptides of the secreted procollagen are cleaved off by extracellular peptidases (**f**) and the processed, mature collagen molecules form a collagen network that is crosslinked and mineralized. Several collagenous and non-collagenous proteins interact with type I collagen to form the bone extracellular matrix (ECM) (**g**). Genes, in which mutations have been linked to osteogenesis imperfecta or related diseases, are indicated. These genes are involved in signal transduction and gene expression (grey), translation (brown), post-translational modification (red), ER homeostasis (purple), proteolytic processing (blue) or ECM structure, and mineralization (green). *MESD is an ER-resident chaperone for LRP proteins, co-receptors of the WNT signaling pathway. ^#^SPARC can act as a chaperone in the ER as well as support mineralization of the collagen matrix extracellularly

In recent years, other genes have been identified causing a clinical picture of OI. Table 1 provides a list of these genes together with the encoded proteins that are changed as well as the mode of inheritance. The molecular targets of most OI types within the maintenance of bone homeostasis are shown in Fig. 1. The figure also displays which cellular and extracellular processes are impaired by mutations in the different genes (signal transduction and gene expression, translation, post-translational modification, ER homeostasis, proteolytic processing, or ECM structure and mineralization).

**Table 1:** A list of these genes together with the encoded proteins that are changed as well as the mode of inheritance

Gene	OMIM gene	Protein	Phenotype	OMIM phenotype
*Autosomal-dominant inheritance*				
*COL1A1*	120150	Collagen α1(I) chain (COL1A1)	OI type I OI type II OI type III OI type IV	166200 166210 259420 166220
*COL1A2*	120160	Collagen α2(I) chain (COL1A2)		
*IFITM5*	614757	Interferon-induced transmembrane protein 5 (IFITM5) *Alternative*: Bone-restricted interferon-induced transmembrane protein-like protein (BRIL)	OI type V	610967
*P4HB*	176790	Protein disulfide-isomerase (PDI) *alternative*: Prolyl 4-hydroxylase subunit beta (P4HB)	Cole-Carpenter syndrome type 1	112240
*Autosomal-recessive inheritance*				
*SERPINF1*	172860	Pigment epithelium-derived factor (PEDF)	OI type VI	613982
*CRTAP*	605497	Cartilage-associated protein (CRTAP)	OI type VII	610682
P3H1	610339	Prolyl 3-hydroxylase 1 (P3H1)	P3H1	610339
*PPIB*	123841	Peptidyl-prolyl cis-trans isomerase B (PPIB) *Alternative*: Cyclophilin B	OI type IX	259440
*SERPINH1*	600943	Serpin peptidase inhibitor, clade H, member 1 (Serpin H1) *Alternative*: Heat shock protein 47 (HSP47)	OI type X	613848
*FKBP10*	607063	Peptidyl-prolyl cis-trans isomerase FKBP10 (PPIase FKBP10) *Alternative:* 65 kDa FK506-binding protein (FKBP65)	Bruck syndrome type 1 OI type XI	259450 610968
*SP7*	606633	Transcription factor Sp7 *Alternative:* Zinc finger protein osterix	OI type XII	613849
*BMP1*	112264	Bone morphogenetic protein 1 (BMP1)	OI type XIII	614856
*TMEM38B*	611236	Trimeric intracellular cation channel type B (TRIC-B) *Alternative*: Transmembrane protein 38B (TMEM38B)	OI type XIV	615066
*WNT1*	164820	Proto-oncogene Wnt1 (wingless-type MMTV integration site family, member 1, WNT1)	OI type XV	615220
*CREB3L1*	616215	Cyclic AMP-responsive element-binding protein 3-like protein 1 (CR3L1)	OI type XVI	616229
*SPARC*	182120	Secreted protein acidic and rich in cysteine (SPARC)	OI type XVII	616507
*TENT5A*	611357	Terminal nucleotidyltransferase 5A (TENT5A) *Alternative:* Family with sequence similarity 46, member A (FAM46A)	OI type XVIII	617952
*MESD*	607783	Mesoderm development LRP chaperone (MESD)	OI type XX	618644
*PLOD2*	601865	Procollagen-lysine,2-oxoglutarate 5-dioxygenase 2 (PLOD2) *Alternative*: Lysyl hydroxylase 2 (LH2)	Bruck syndrome type 2	609220
*SEC24D*	607186	Protein transport protein Sec24D (SEC24D) *Alternative*: SEC24-related protein D	Cole-Carpenter syndrome type 2	616294
*X-linked inheritance*				
*MBTPS2*	300294	Membrane-bound transcription factor site-2 protease (MBTPS2) *Alternative*: Endopeptidase S2P	OI type XIX	301014
*PLS3*	300131	Plastin-3 (PLS3)	Osteoporosis (X-linked dominant)	300910

### Collagen biosynthesis, modification, and secretion

Mutations in several genes can result in the clinical picture of OI without changing the collagen sequences but rather affecting the biosynthetic pathway and secretion of collagens. This process involves multiple steps and requires a large number of proteins for post-translational modification, folding, transport, and quality control of collagen type I.

#### P4HB

The helical region of the collagen molecule is rich in proline residues, which are arranged in repetitive sequences. After translating the procollagen into the rough endoplasmic reticulum (ER), one of the first steps in the post-translational modification is the hydroxylation of proline residues in the helical region to hydroxyproline. The hydroxylation leads to greater stability of the collagen triple helix. The prolyl-4-hydroxylation is performed by the tetrameric prolyl-4-hydroxylase complex. A subunit of this complex, the protein PDI (protein disulfide isomerase), is encoded by the *P4HB* gene. PDI serves as a molecular chaperone and regulates disulfide bridge formation. However, the pathomechanism of the identified *P4HB* mutations is not yet known. Mutations lead to type 1 Cole Carpenter syndrome, but are also clinically described as moderate to severe OI [5].

#### P3H1, CRTAP, and PPIB

Prolyl-3-hydroxylation on specific proline residues in the collagen molecule is carried out by three different prolyl-3-hydroxylase isoforms. The complex consisting of P3H1 (prolyl-3-hydroxylase 1), CRTAP (cartilage-associated protein), and PPIB (peptidyl-prolyl-cis-trans-isomerase B or cyclophilin B) is responsible for the hydroxylation of proline-986 in the α1 chain. Recessive mutations in the three respective genes (*P3H1*, *CRTAP*, and *PPIB*) cause OI of different severities. Mutations lead to a decrease in proline-986 hydroxylation and thus to a delay in collagen folding, accompanied by an excessive modification [6]. Intracellular retention and aggregation of overmodified collagen can lead to ER stress and induce cell death. In the process of hydroxylation, cyclophilin B (=PPIB) ensures the cis-trans isomerization of the collagen-prolyl-peptide bond and, together with FKBP65 (also known as FKBP10), a molecular chaperone, prevents the procollagen chains from being assembled into fibrils prematurely. Cyclophilin B can also interact with the lysyl hydroxylase 1 (LH1), thus influencing lysyl hydroxylation of the collagen chains and intermolecular crosslinking [7].

Mutations in *CRTAP* tend to trigger more severe forms of the disease. Most children have fractures of the long bones before or immediately after birth. Most adults depend on a wheelchair or other aids for mobility. Mutations in *P3H1* are often described as causing equally severe phenotypes. However, there are also patients who are moderately affected, without prenatal fractures, most of which can walk as they get older. Patients with PPIB mutations are often severely, rarely moderately affected [8].

#### PLOD2

*PLOD2* encodes the protein lysyl hydroxylase 2 (LH2), which similar to lysyl hydroxylase 1 (LH1), hydroxylates lysine residues in the collagen molecule. Hydroxylation of proteins enables covalent crosslinking within the molecule and thus imparts tensile strength. Hydroxylysines can also be glycosylated. Although the exact function of collagen glycosylation is not yet fully understood, it is essential for the stability of collagen. Mutations in the *PLOD2* gene cause Bruck syndrome type 2, which is characterized by moderate to severe skeletal changes and progressive joint contractures with OI type XI [9]. With regard to the fragility of the bones, it is more of a moderate OI phenotype, but the progressive contractures are decisive for the quality of life. It has recently been shown that the activity of LH2 is regulated by the molecular chaperones HSP47 (encoded by the gene *SERPINH1*) and FKBP65 (encoded by *FKBP10*) in the ER.

#### SERPINH1

Heat shock proteins belong to the family of molecular chaperones and prevent the aggregation of protein folding, but are also involved in the association of collagen chains with superordinate fibrillar structures. The gene *SERPINH1* encodes for the chaperone HSP47 (heat shock protein 47), and mutations in this gene lead to misfolding and/or instability of the protein. This results in delayed collagen secretion as well as changed collagen structure or partial retention of the collagen within the cell [10]. Mutations in *SERPINH1* cause moderate and severe forms of OI, some with severe deformities and prenatal fractures, but some with fractures only in the first months of life.

#### FKBP10

The gene *FKBP10* encodes the chaperone FKBP65 (65-kDa FK506-binding protein 10). Mutations can lead to Bruck syndrome type 1 (congenital contractures) with more or less severe brittleness. It can also cause a severe phenotype of OI without contractures. Although the collagen structure appears to be normal, changes in collagen stability have been described. This causes an accumulation of procollagen aggregates in the ER. In addition, the intermolecular collagen linkage is markedly reduced, comparable to patients with a mutation in *PLOD2* [5]. This phenotypic overlap indicates a functional interaction between the two proteins.

#### TMEM38B

*TMEM38B* (Transmembrane Protein 38B) is a gene coding for a monovalent cation channel (TRIC-B, trimeric intracellular cation channel type B). This ER membrane-integral potassium channel is necessary for emptying intracellular calcium stores and plays a role in cell differentiation. A disturbed intracellular calcium release leads to an incorrect regulation of collagen modification by various enzymes in the ER. This results in ER stress and reduced collagen secretion. Mutations in this transmembrane protein are inherited in an autosomal recessive manner and are associated with a moderate form of OI without blue-colored sclerae, dentinogenesis imperfecta, or hearing impairment [5].

#### MBTPS2

*MBTPS2* is an X-linked gene coding for a membrane-bound zinc metalloprotease (S2P, site-2 protease). This protease is associated with various intracellular signaling cascades, including regulated intramembrane proteolysis (RIP) of the transcription factors CR3L1, ATF6, and SREBP. Mediated by a reduced amount of LH1, which leads to a reduced hydroxylation of a lysine residue and a disturbed collagen cross-linking, there is a reduced collagen secretion as well as an impaired differentiation of osteoblasts. Only a few patients have been identified carrying this mutation and present with a moderate to severe OI phenotype [11].

#### CREB3L1

*CREB3L1* (cyclic AMP-responsive element-binding protein 3-like protein 1) encodes a transcription factor (CR3L1, formerly OASIS). Upon ER stress, the N-terminal fragment of CR3L1 that contains a transcription factor is released by two sequentially acting metalloproteases (S1P, S2P (see *MBTPS2*)) to induce the expression of the unfolded protein response (UPR) genes. Binding of the UPR element-like sequence of CR3L1 activates the osteoblast-specific *COL1A1* promoter region that does not exist in the corresponding skin-specific *COL1A1* promoter region. Therefore, mutations result in reduced collagen production in bone but not in the skin cells of affected patients. This is partly accompanied by an altered composition and hypermineralization of the bone matrix. Patients with a biallelic mutation usually present a moderate to a severe clinical course, often with prenatal fractures and shortening of the long bones [12]. Humans with a heterozygous genotype are more mildly affected, with fractures only after birth, and most are able to walk independently. Some of the heterozygous individuals do not even show clinical symptoms of a skeletal disease, i.e., they are asymptomatic carriers for the severe autosomal recessively inherited OI phenotype.

#### SEC24D

*SEC24D* encodes a protein of the COPII-dependent ER-to-Golgi transport. A mutation in this gene leads to molecular retention of procollagen in the ER and clinically causes a disturbed ossification of the skull bones with craniofacial malformations and an increased fracture rate, sometimes with a prenatal onset. This symptom constellation is called Cole-Carpenter syndrome type 2 [13]. Other patients carrying mutations in this gene have been clinically diagnosed with OI, including a classic OI type with the gray-blue sclera, wormian bones, and shortened or bent long bones. Most patients are able to walk and participate in life almost normally.

#### SPARC

Two missense mutations have been described in the *SPARC* gene so far. These lead to an exchange of amino acids in the SPARC protein (secreted protein, acidic and rich in cysteine, alternatively referred to as osteonectin/BM-40) which are essential for the binding between SPARC and collagen. Intracellularly, SPARC can serve as a molecular chaperone during collagen biosynthesis. Accordingly, slight overmodification and delayed secretion of collagen were observed in patient cells. In the extracellular space, SPARC mediates extracellular matrix-cell interactions and promotes mineralization of the extracellular matrix by binding to collagen and hydroxyapatite. Thus, SPARC fulfills multiple roles in maintaining bone mass and quality. The two first described patients with SPARC mutations are moderately affected [14].

#### BMP1

After the secretion of collagen into the extracellular space, the proteolytic cleavage of propeptides is necessary to enable collagen assembly and fibril formation. Mutations in the *BMP1* gene, which codes for the protease BMP1 (bone morphogenetic protein 1) responsible for the extracellular cleavage of C-propeptides, result in deficient proteolytic cleavage and a very variable phenotype ranging from mild to severe. Procollagen processing and the ability to generate mature collagen fibrils are limited in cells of these patients. This leads to an increased mineralization of the collagen matrix and increased bone mass [15]. Interestingly, mutations affecting the BMP1 cleavage site in both collagen type I α chains also lead to a mild type of OI, characterized by increased bone mass [16].

### Impairment of osteoblast differentiation and function

Recently, some further genes have been identified which affect the differentiation as well as the function of osteoblasts and are thus important for the stability of the skeletal system. For most of them, the underlying pathomechanism caused by mutations in these genes is not yet fully elucidated.

#### SP7

Mutations in *SP7*, which encodes the osteoblast-specific transcription factor SP7 (or osterix) and initiates the differentiation of pre-osteoblasts into osteoblasts as well as osteocytes, lead to a rather mild instability of the bones with repeated fractures. Patients with mutations in *SP7* are sometimes affected by an early hearing loss. These patients show increased bone porosity, which could possibly be attributed to increased trabecular bone remodeling due to impaired balance between bone formation by osteoblasts and bone resorption by osteoclasts [17].

#### WNT1

Mutations in this gene encoding the secreted glycoprotein WNT1 (wingless-type MMTV integration site family 1), which induces the WNT signaling pathway, lead to OI with a very heterogeneous clinical severity. After binding of WNT1 to the dual receptor complex of Frizzled and LRP5/6 (low-density lipoprotein receptor-related protein 5/6), the second messenger β-catenin is stabilized and translocates to the nucleus. Here, it induces the expression of genes regulating osteoblast differentiation and function. Mutations in the *WNT1* gene result in altered signal transduction and restricted expression of osteoblast-specific genes regulating bone cell homeostasis. Despite normal bone mineralization, patients with *WNT1* mutations show reduced bone remodeling, indicating an imbalance between bone formation and resorption [18]. In mouse models, the lack of functional WNT1 in osteoblasts has been shown to be responsible for the manifestation of OI. Since WNT1 is also expressed in the brain, patients are often co-affected by developmental disorders of the central nervous system and display a varying degree of cognitive impairment. Interestingly, mutations in the WNT1 co-receptor LRP5 lead to the osteoporosis pseudoglioma syndrome with overlapping bone features to OI.

#### MESD

*MESD* (mesoderm development gene, previously referred to as *MESDC2*) encodes an ER chaperone for the canonical WNT signaling receptors LRP5 and LRP6. In at least five independent families autosomal recessive mutations in *MESD* have been identified. These OI-causing homozygous truncation or frameshift mutations occur downstream of the chaperone activity domain but upstream of the ER-retention signal peptide and produce hypomorphic alleles. Patients with *MESD* mutations suffer from a progressively deforming type of OI with recurrent fractures, and one patient was reported with oligodontia. Interestingly, skeletal fragility or oligodontia also occurs in individuals deficient for LRP5 or LRP6, respectively. However, OI-causing mutations in *MESD* do not fully phenocopy humans or mice that are completely deficient for either LRP5 or LRP6. With regard to the pathomechanism, it is therefore believed that *MESD* mutations reduce but do not completely abolish the chaperone function for LRP5 and LRP6 [19].

#### TENT5A

Recently, mutations in the gene *TENT5A* (formerly known as *FAM46A*), encoding the terminal nucleotidyltransferase 5A were described as disease-causing in three patients. These patients are moderately to severely affected [20]. The expression of TENT5A in osteoblasts suggests a role in bone homeostasis and a previously unknown function of this enzyme in mineralized tissue was described. This theory is supported by a new mouse model with skeletal dysplasia. These mice carry a Tent5a mutation and develop an OI-like phenotype, but the pathomechanism behind it has not yet been elucidated. Studies in the clawed frog (Xenopus) show that the TENT5A protein activates the BMP signaling pathway essential for bone formation and homeostasis by stabilizing the effector SMAD1.

### Further genes influencing bone stability

Mutations in three genes that have been linked to OI are implicated in the regulation of extracellular matrix mineralization or osteoclast function. In contrast to the bone-forming osteoblasts, osteoclasts resorb bone material and a disturbed balance of bone formation and resorption inevitably will result in defective bone homeostasis.

#### IFITM 5

*IFITM5* encodes the protein of the same name (interferon-induced transmembrane protein-5, formerly BRIL (bone-restricted interferon-induced transmembrane protein-like protein)). The function of IFITM5, and even more the pathomechanism of the changes triggered by the known mutations, is still not fully understood. IFITM5 seems to play a role in osteoblast differentiation and bone mineralization. In osteoblast cultures, a delay in differentiation, a reduced amount of collagen, and an increased mineralization could be shown. The first known and most common mutation in humans (5′-non-translated region, new start codon), which leads to a gain-of-function, is responsible for the typical OI type V symptoms. These include hyperplastic callus formation after fractures and excessive ossification of the membrana interossea in the forearms [21]. In addition, there are mutations in the coding region of *IFITM5* that lead to reduced mineralization, similar to established mouse models. Furthermore, there is a so far unclear interaction between IFITM5 and PEDF, which was observed in patients with a non-classical IFITM5 mutation, but was clinically similar to OI type VI.

#### SERPINF1

The *SERPINF1* gene plays a special role among the recessive genes. Mutations in this gene do not affect collagen formation or differentiation of osteoblasts, but increase bone resorption. *SERPINF1* encodes the protein pigment epithelium-derived factor (PEDF) that induces the expression of osteoprotegerin, a physiological inhibitor of osteoclastogenesis through blockade of RANKL. A loss-of-function mutation in *SERPINF1* leads to an increased differentiation and activation of osteoclasts, mediated by the misregulated RANKL/osteoprotegerin system. Thus, an increased degradation of bone mass takes place [22]. Patients usually do not have perinatal fractures, and first fractures do not occur until 4–18 months of age. The frequency of fractures and the severity of the disease are progressive. The sclerae and teeth are usually not affected. The reported overactivation of osteoclasts led to a new therapeutic approach, which allows a more targeted treatment based on the pathophysiology. The RANKL-antibody denosumab is approved in adults with osteoporosis and showed also a beneficial effect in children with OI caused by mutations in *SERPINF1* [23].

#### PLS3

*PLS3* is coding for the cytoskeletal protein plastin-3 that is ubiquitously expressed, including in all bone cell types, and appears to play a role in bone formation, mineralization, and bone resorption. Plastin-3 is involved in the formation of F-actin bundles by binding to F-actin and the regulation of the NF-κB pathway. It interacts with the NF-κB repressing factor (NKRF) and NKRF binds to the NFκB downstream target and master regulator of osteoclastogenesis nuclear factor of activated T cells 1 (NFATc1), thereby reducing its transcription and suppressing osteoclast function. Mutations can lead to early manifesting osteoporosis, or they can cause an OI-like phenotype in severe cases. Due to the X-linked chromosomal inheritance, male (hemizygous) patients are usually earlier and more severely affected by osteoporosis than female (heterozygous) individuals (which may be asymptomatic). However, within the OI spectrum, most affected males would be classified as having mild OI. Pathomechanistically, an altered mechanotransduction is assumed due to the disturbed interaction of the extracellular matrix with the cytoskeleton of bone cells, primarily with the osteocytes. Furthermore, the resorptive activity, adhesion, and migration of osteoclasts are directly dependent on the formation of large actin filaments. Mutations in *PLS3* lead to a reduced trabecular thickness with normal expression and modification of collagen as well as to a normal pattern of bone lamellar structure. This change, like other clinical and laboratory findings, suggests the overactivity of osteoclasts as a partial reason for the bone phenotype [24, 25].

### Medical treatment

Medical treatments with bisphosphonates are currently used as standard therapy in patients with a moderate or severe course in childhood and adolescence. Upon administration bisphosphonates bind to the hydroxyapatite crystals of the bone, which are resorbed by osteoclasts during bone remodeling and induce their apoptosis. These drugs effectively reduce bone resorption and thereby increase bone mass. Bisphosphonates are approved for the therapy of geriatric osteoporosis and dosing and treatment intervals are adapted to the pediatric needs. It has been shown that intravenous therapy has a positive effect on skeletal pain and bone mass, and in addition, mobility of patients can be improved [26]. Different bisphosphonates (pamidronate, neridronate, zoledronate) have also been used and differ in treatment intervals. Oral bisphosphonates are less effective but can also be used in special indications. Treatment must be carried out regularly and should continue during growth [27]. Despite a broad consent about the beneficial effect of bisphosphonates in moderate or severely affected children a reduction of fractures was never shown in this population [28]. It has to be mentioned that bisphosphonates are not approved for children in Germany and many other countries and can only be administered as “off-lable-use” after careful discussions and written consent.

### Surgical treatment

Orthopedic procedures have two main objectives in the treatment concept of patients with OI—to reduce pain and ensure healing without an increase of deformities. In case of fractures without dislocation or in patients in which the bone is supported by an intramedullar rod, conservative treatment with a cast is often sufficient. In severely affected children with deformities of the long bones, which might prevent verticalization of the patient or restrict the functional use of the upper extremity surgical treatment with correction of deformities is indicated. During growth, intramedullar telescopic rods should be inserted. These elongate during growth and can support the bones for many years [29].

It should be noted that fractures in patients with classical OI caused by mutations in COL1A1/2 heal just as quickly as in non-affected individuals, and therefore, prolonged immobilization is not required. In some rare types, healing might be altered. For example, this might occur in patients with mutations in IFITM5 (hyperplastic callus formation) or WNT1 (delayed healing). A long immobilization needs to be avoided in order to prevent muscle wasting and consequently resorption of bone mass due to immobilization.

### Physiotherapy

Medical and surgical treatments aim to improve the quality of life, mobility, and independence of patients. Many patients are also affected by a muscular hypotonia and weakness of tendons and ligaments. Regular strengthening of the muscles is crucial to improve mobility. In addition, children have to learn which motor functions are possible after surgery. If deformities of the long bones have been corrected at the age of 2–3 years, the older child has then to learn weightbearing and standing, and it has to be determined which assistive devices are suitable to improve mobility.

Because of recurrent fractures, many patients are afraid to try new movement patterns, which must be taken into account during training. In addition, the strengthening of muscles induces an osteoanabolic stimulus, which leads to an increase in the synthesis of extracellular matrix by osteoblasts. Although the function of osteoblasts can be impaired in OI, using the muscles is still the best way to stimulate bone formation [30].

## Data Availability

Not applicable

## Abbreviations

BMP1: Bone morphogenetic protein 1
BRIL: Bone-restricted interferon-induced transmembrane protein
COL1A: Collagen α1 chain
COP II: Coat protein complex II
CREB3L1: Cyclic AMP-responsive element-binding protein 3-like protein 1
CRTAP: Cartilage-associated protein
ECM: Bone extracellular matrix
ER: Endoplasmic reticulum
HSP47: Heat shock protein 47
IFITM5: Interferon-induced transmembrane protein-5
LH1: Lysyl hydroxylase 1
LH2: Lysyl hydroxylase 2
LRP5/6: Low-density lipoprotein receptor-related protein 5/6
MESD: Mesoderm development gene
NKRF: NF-κB repressing factor
OI: Osteogenesis imperfecta
P3H1: Prolyl-3-hydroxylase 1
P4HB: Prolyl 4-hydroxylase subunit beta
PDI: Protein disulfide isomerase
PEDF: Pigment epithelium-derived factor
PPIB: Peptidyl-prolyl-cis-transisomerase B
RIP: Intramembrane proteolysis protein 1
S2P: Site-2 protease)
SEC24D: Protein transport protein Sec24D
Serpin H1: Serpin peptidase inhibitor, clade H, member 1
SPARC: Secreted protein, acidic and rich in cysteine
TENT5A: Terminal nucleotidyltransferase 5A
TMEM38B: Transmembrane protein 38B
TRIC-B: Trimeric intracellular cation channel type B
UPR: Unfolded protein response
WNT1: Wingless-type MMTV integration site family 1
